# Assessment of Ovarian Tumor Growth in Wild-Type and Lumican-Deficient Mice: Insights Using Infrared Spectral Imaging, Histopathology, and Immunohistochemistry

**DOI:** 10.3390/cancers13235950

**Published:** 2021-11-26

**Authors:** Pierre Nizet, Valérie Untereiner, Ganesh D. Sockalingum, Isabelle Proult, Christine Terryn, Albin Jeanne, Lise Nannan, Camille Boulagnon-Rombi, Christèle Sellier, Romain Rivet, Laurent Ramont, Stéphane Brézillon

**Affiliations:** 1 Laboratoire de Biochimie Médicale et Biologie Moléculaire, UFR Médecine, Université de Reims Champagne-Ardenne, 51097 Reims, France; pierre.nizet@univ-reims.fr (P.N.); isabelle.proult@univ-reims.fr (I.P.); a.jeanne@apmonia-therapeutics.com (A.J.); lise.nannan1@univ-reims.fr (L.N.); christelle.sellier@univ-reims.fr (C.S.); romain.rivet@univ-reims.fr (R.R.); lramont@chu-reims.fr (L.R.); 2 CNRS UMR 7369, Matrice Extracellulaire et Dynamique Cellulaire-MEDyC, 51097 Reims, France; cboulagnon-rombi@chu-reims.fr; 3 Université de Reims Champagne-Ardenne, PICT, 51097 Reims, France; valerie.untereiner@univ-reims.fr (V.U.); christine.terryn@univ-reims.fr (C.T.); 4 Université de Reims Champagne-Ardenne, BioSpecT-EA7506, UFR Pharmacie, 51097 Reims, France; 5 Apmonia Therapeutics SAS, CREA, 2 esplanade Roland Garros, 51686 Reims, France; 6 CHU Reims, Service de Pathologie, 51092 Reims, France; 7 CHU Reims, Service de Biochimie-Pharmacologie-Toxicologie, 51092 Reims, France

**Keywords:** ovarian cancer, lumican, collagen, infrared imaging, glycosaminoglycan, immunohistochemistry, second harmonic generation, K-means clustering

## Abstract

**Simple Summary:**

Lumican, a small leucine-rich proteoglycan (SLRP), maintains extracellular matrix (ECM) integrity while inhibiting melanoma primary tumor development, as well as metastatic spread. The aim of this study was to analyze the effect of lumican on tumor growth of murine ovarian carcinoma. C57BL/6 wild type mice (*n* = 12) and lumican-deficient mice (*n* = 10) were subcutaneously injected with murine ovarian epithelial carcinoma ID8 cells, and sacrificed after 18 days. Label-free infrared spectral imaging (IRSI) generated high contrast IR images allowing identification of different ECM regions of the skin and the ovarian tumor. IRSI showed a good correlation with collagen distribution as well as organization, as analyzed using second harmonic generation imaging within the tumor area. The results demonstrated that lumican inhibited the growth of ovarian cancer mainly by altering collagen fibrilogenesis.

**Abstract:**

Ovarian cancer remains one of the most fatal cancers due to a lack of robust screening methods of detection at early stages. Extracellular matrix (ECM) mediates interactions between cancer cells and their microenvironment via specific molecules. Lumican, a small leucine-rich proteoglycan (SLRP), maintains ECM integrity and inhibits both melanoma primary tumor development, as well as metastatic spread. The aim of this study was to analyze the effect of lumican on tumor growth of murine ovarian epithelial cancer. C57BL/6 wild type mice (*n* = 12) and lumican-deficient mice (*n* = 10) were subcutaneously injected with murine ovarian epithelial carcinoma ID8 cells, and then sacrificed after 18 days. Analysis of tumor volumes demonstrated an inhibitory effect of endogenous lumican on ovarian tumor growth. The ovarian primary tumors were subjected to histological and immunohistochemical staining using anti-lumican, anti-αv integrin, anti-CD31 and anti-cyclin D1 antibodies, and then further examined by label-free infrared spectral imaging (IRSI), second harmonic generation (SHG) and Picrosirius red staining. The IR tissue images allowed for the identification of different ECM tissue regions of the skin and the ovarian tumor. Moreover, IRSI showed a good correlation with αv integrin immunostaining and collagen organization within the tumor. Our results demonstrate that lumican inhibits ovarian cancer growth mainly by altering collagen fibrilogenesis.

## 1. Introduction

Ovarian cancer is the gynecological malignancy with the highest case-to-mortality ratio in the western world. Since ovarian cancer is often asymptomatic, it is generally diagnosed at an advanced stage, giving a poor prognosis [1]. It is the second leading cause of death among patients with gynecologic tumors in the world, as 313,959 women were diagnosed with ovarian cancer, and 207,252 (66%) died of it in 2020 [2]. Most tumors initially respond to standard treatments combining surgery and platinum-based chemotherapy. Moreover, novel second line treatments and maintenance therapies (such as PARP inhibitors and anti-angiogenic antibodies) permit an improvement in survival and patient welfare [3]. However, frequent recurrence, subsequent acquired chemoresistance, and widespread dissemination are responsible for the therapeutic ineffectiveness, leading to an overall 10-year survival rate of 35% [4]. Ovarian cancers usually start within the ovary (or ovaries) or the fallopian tubes, before spreading to other organs in the pelvic region, then finally metastasizing to peritoneal organs. The mesothelium, a single layer of flat cells covering the peritoneal cavity and its organs, is the first barrier met by ovarian tumor cells, and is the major site of ovarian carcinoma metastasis before invading the underlying connective tissue rich in fibroblasts. Due to the rapid proliferation and spread of ovarian cancer within the abdominal cavity and its high rate of intra-abdominal recurrence, the prognosis of patients with this condition is poor. Recently, electronic microscopy permitted a better discrimination of human ovarian cancer cells heterogeneity [5].

The most common epithelial ovarian cancer (EOC) histological subtype, accounting for >50% of ovarian epithelial malignancies, is serous ovarian carcinoma [6,7]. Due to the lack of early detection tools, most serous ovarian carcinomas (>80%) are diagnosed at a more advanced stage (stages III and IV), where the 5-year survival rate remains at only 34% and 15%, respectively [4]. Most of these (>50%) are classified as “high-grade“ tumors, based on their degree of nuclear atypia and high mitotic index [8]. High-grade serous ovarian carcinomas (HGSOC type II) are characterized from other subtypes both by their aggressive nature and the unique genetic alterations they may harbor, including TP53 and the homologous recombination (HR) DNA repair genes BRCA 1 and 2. Tumors mutated from these HR genes are usually more receptive to chemotherapy, due to their inability to correctly repair their DNA [9]. In contrast, clear cell, endometrioid, low-grade serous, and mucinous ovarian carcinomas are typically present as indolent low-grade neoplasms (type I tumors) with somatic mutations in genes such as KRAS, BRAF, ERBB2, PTEN, CTNNB1, and PIK3CA [10]. Most ovarian cancer cell lines are of human origin. Indeed, to our knowledge, ID8 is the only well described murine ovarian cancer model. ID8 represents a cell line derived from spontaneous malignant transformation of C57BL/6 MOSEC (mouse ovarian surface epithelial cells) in vitro [11]. Many of the clinical features typical of ovarian cancer, including the presence of tumor nodules throughout the omentum and lymphogenic metastasis in the lungs, were seen in this model, as was the formation of hemorrhagic ascites [12]. This model was recently used to demonstrate the role of TAX2 peptide, a drug candidate under development, to activate anti-tumor immunity [13].

In multicellular organisms, cells are surrounded by a crowded extracellular environment, either a complex architectural extracellular matrix (ECM) network or a liquid environment. The tumor microenvironment is composed of various cell types embedded in an altered ECM. The latter not only serves as a support for tumor cells, but also regulates cell–cell or cell–matrix cross-talks. Tumor ECM is essential to tumor progression, and ECM alterations are often associated with a poorer prognostic for patients. Tumor ECM proteome is strongly altered, and different ECM protein signatures may serve as prognostic biomarkers. Collagen network reorganization facilitates tumor cell invasion. Proteoglycan expression and location are modified in the tumor microenvironment, and affect cell invasion and metastatic dissemination [14].

Lumican belongs to the small leucine-rich proteoglycans (SLRP) family [15,16] and was shown to control the assembly of collagen fibers in the ECM [17,18]. Amino acid sequence data of lumican (LUM) indicate that the central region of the protein contains four asparagine residues that are N-linked with keratan sulphate (KS) or oligosaccharides [19,20]. The molecular mass of the core protein is 38 kDa, and can increase to 55–57 kDa in the glycoprotein form, and 50–100 kDa, or even higher, in the proteoglycan form [21]. Several forms of LUM are differentially expressed in tissue. The non-glycosylated form of LUM was observed in lung fibroblasts [22]. The glycoprotein form was detected in the dermis [21,23,24,25], and the KS form of LUM was found in corneal stroma [26]. Lumican is expressed in various tumor tissues, but both positive and negative correlations with tumor aggressiveness have been reported [25,27,28], highlighting it as either a therapeutic molecule or an anti-cancer target. In breast tumors, the expression of lumican was detected at the mRNA and protein levels, and it was concluded that lumican is the most important proteoglycan in breast tumors [29]. Lumican was identified in human ovarian cancer ascites [30], where it was more abundant, as compared to serum control samples [31]. LUM and decorin (DCN) showed reduced stromal expression in serous epithelial ovarian cancer [32]. The expression of lumican in cytostatic-resistant ovarian cancer tissue suggests that it might also have a role in drug resistance. Moreover, the expression of lumican in this context is correlated with the expression of the alpha-1 chain of type III collagen [33].

Second harmonic generation (SHG) and Fourier transform infrared (FTIR) imaging techniques were used to evidence ECM disorganization by lumican in melanoma and, more specifically, collagen fiber orientation [34]. SHG is commonly used to assess the structure and abundance of collagen fibrils in a non-invasive, highly resolutive and specific process [35]. IR spectroscopy is used for structural and compositional analysis due to its ability to give a complete “molecular fingerprint” of the sample [36]. It has previously been used to characterize ovarian cancer cells and tissues [37]. At the tissue level, FTIR imaging, combined with multivariate statistical analysis, has shown the ability to discriminate inflammatory from non-inflammatory breast cancer tissues [38], and metastatic from non-metastatic lymph nodes in melanoma patients [39]. In a recent study, dermis of wild-type versus lumican-deficient mice were characterized by infrared spectral imaging [40]. In addition, melanoma primary tumors treated with or without lumican-derived peptide were discriminated through infrared spectral imaging [41].

In the present report, we investigated the effect of lumican on ECM organization and tumor progression in ovarian primary tumors by combining FTIR and SHG imaging with conventional histology and immunohistochemistry. Altogether, both imaging and histological methods evidence that the absence of LUM leads to a loss of ECM integrity by disorganization of the collagen fiber network, potentializing edema formation and tumor progression.

## 2. Materials and Methods

**Figure 1:**
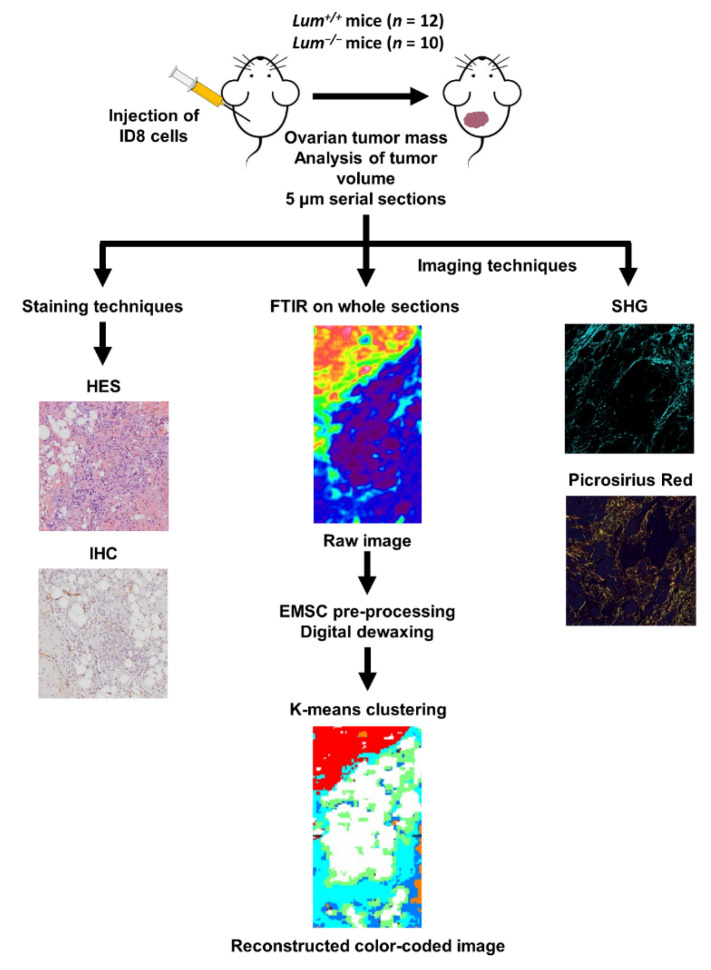
Workflow showing the histology, the immunohistochemistry of formalin-fixed paraffin-embedded ID8 ovarian tumor sections, SHG imaging, Picrosirius red staining (polarized light), and analysis of FTIR images using common K-means clustering.

The workflow for histopathological, immunohistochemical, label-free infrared spectral imaging (IRSI) and SHG analyses is illustrated in Figure 1.

### 2.1. Cell Culture

The ID8 cell line (murine ovarian epithelial carcinoma) was purchased from Sigma-Aldrich, St. Louis, MO, USA, and cultured in DMEM medium (Sigma-Aldrich) supplemented with 4% FCS (PAN Biotech, Aidenbach, Germany), 100 µg/mL streptomycin, 100 U/mL penicillin, 5 µg/mL insulin transferrin mix, and 5 ng/mL sodium selenite (Sigma-Aldrich). All cell cultures were performed in a 95% humidified atmosphere with 5% CO_2_ at 37 °C.

### 2.2. Animal Care

The *Lum^−/−^* mouse line was generated by targeted mutation and fixed to the C57BL/6J genetic background (B6.129S-Lumtm1Chak/J) [19]. PCR-based genotyping was performed to distinguish between homozygous *Lum^−/−^* mice and their wild-type (WT, i.e., *Lum^+/+^*) littermates. A mixture of three primers (forward primer, 1893U: 5′-AAG CAG GGG ATG TTA AGC TGC-3′, reverse primers 2187: 5′-ACG TGC TAC TTC CAT TTG TCA CG-3 and 2231L: 5′-TCA GGG TAT TTC CTG GTG GCA C-3) was used to amplify a 338 bp and a 294 bp products from wild type and lumican deficient mice, respectively. The mice body weight was measured every week. The mice clinical status and behavior were controlled daily.

### 2.3. Allograft Model

The ID8 cell line was maintained in culture as reported above. For allograft experiments, 2.5 × 10^5^ ID8 cells suspended in 100 μL of DMEM medium (Sigma-Aldrich) were subcutaneously (s.c.) inoculated into the left flank of randomized groups of either WT (*Lum^+/+^*) or lumican-deficient (*Lum^−/−^*) mice (*n* = 10–12 per group). On day 8, tumors were detectable, and tumor volume was measured every 1–2 days. Tumor measurements and animal monitoring were performed as reported elsewhere [23,42,43,44]. On day 18, mice were sacrificed, and tumors were surgically extracted, weighted, and then fixed in 4% formaldehyde.

### 2.4. Histopathological Analyses

Histological analyses of formalin-fixed paraffin-embedded (FFPE) s.c. allografts were performed on hematoxylin, eosin, and saffron (HES)-stained sections, prepared using routine histological methods. Picrosirius red staining was performed to observe the birefringence of collagen fibers to distinguish between type I (red) and type (III) (green) collagens under polarized light (see Section 2.8). All stained sections were digitized with the VS120 digital scanner (Olympus, Tokyo, Japan).

The rabbit polyclonal antibody raised against lumican core protein [23,34] (1:1600), the rabbit anti-cyclin D1 (1:200, #RM-9104-SO, Thermo Fisher Scientific, Waltham, MA, USA), the rat anti-CD31 (1:200, #DIA-310; Dianova GmbH, Hamburg, Germany), and the rabbit anti-αv integrin (1:200, #60896, Cell Signaling, Ozyme, Saint-Cyr-L’École, France) antibodies were used to perform immunostaining together with biotin-labeled secondary antibodies and a streptavidin-HRP AEC (3-amino-9-ethylcarbazole) (# TA-125-SA, ThermoFisher Scientific, Waltham, MA, USA) or a DAB (3,3’-diaminobenzidine) detection system (Abcam), followed by hematoxylin counterstain. Negative controls were performed by omitting the primary antibody. 

The number of functional blood vessels (i.e., vessels displaying endothelial layer integrity) and their mean diameter, as well as relative CD31-positive areas, were determined in whole tumor areas. The number of cyclin D1 positive and negative nuclei were assessed in whole tumor areas, and their ratio was compared between *Lum^+/+^* and *Lum^−/−^* mice. All quantitative analyses were performed using QuPath software [45].

### 2.5. Fourier Transform Infrared (FTIR) Microimaging

For FTIR microimaging, 5 μm FFPE tissue sections (adjacent to HES staining) were transferred onto CaF_2_ windows, and then spectral images were collected using an infrared microscope (Spotlight 400 Imaging System, PerkinElmer, Villebon-sur-Yvette, France), coupled with a Frontier spectrometer. Spectral data were acquired in transmission mode at a pixel size of 6.25 × 6.25 µm^2^, using a spectral resolution of 4 cm^−1^, and averaged to 8 scans per pixel on the spectral range 800 to 4000 cm^−1^. For each sample, an image of pure paraffin was collected under the same conditions, as well as a background spectrum (90 scans) to ratio against the single-beam spectrum. Further multivariate statistical analyses were performed using in-house algorithms written in MATLAB (The Mathworks, Natick, MA, USA). A modified extended multiplicative signal correction (EMSC) method was used to digitally deparaffinize the tissue by neutralizing paraffin variability in each FTIR pixel spectrum [46], as well as to eliminate spectra with low signal-to-noise ratio [47].

### 2.6. FTIR Images Processing by Unsupervised K-Means Clustering

An unsupervised cluster analysis was applied to correct FTIR images using the K-means method [48] on the spectral range 900 to 1800 cm^−1^. K-means clustering iteratively partitions spectra into different classes based on spectral signatures. First, K spectra (K is the predefined number of searched clusters) were randomly chosen to represent initial centroids that model the mean spectrum of each cluster. Second, each spectrum was assigned to the cluster with the nearest centroid based on the Euclidean distance calculation. Third, each centroid was updated as the mean of the spectra belonging to its cluster. Steps 2 and 3 were repeated until convergence was reached. Spectra belonging to the same cluster are represented by the same pseudo-color. All spectra eliminated by EMSC (i.e., pure paraffin and low signal-to-noise ratio spectra) appeared as white pixels.

### 2.7. Correlation of IR Spectral Images with Type I Collagen Spectrum

Spectral images of the tumor sections were correlated with a type I collagen representative spectrum to assess the distribution of collagen. This was performed by first recording a spectrum of type I collagen from a 5 µm thick section of FFPE rat-tail tendon, using the same conditions as for the ovarian tumor tissue sections. This spectrum was then correlated pixel by pixel with the tumor image using the Spectrum Image 6.4 software (PerkinElmer). The correlation process resulted in a new image with a correlation scale ranging from 0 (dark color) to 1 (white color). In this way, the distribution of collagen in the tissue could be visualized [40].

### 2.8. Polarized Light Microscopy

To study ECM collagen organization, deparaffinized tissue sections were stained using the Abcam Picrosirius red stain kit (ab150681), according to the manufacturer’s instructions. Picrosirius red stained tissues were observed using polarized light, resulting in birefringence of the collagen fibers, and allowed distinction between type I (thick fibers, red birefringence) and type III (thin fibers, green birefringence) collagens. Slides were imaged using a VS120 digital scanner (Olympus, Tokyo, Japan) equipped with cross polar optics. For each slide, color channels were split in ImageJ software before thresholding to quantify the percentage of red and green pixels. For each image, mean red and green intensities and their ratio were also calculated, and then averaged for each mouse group.

To assess the basketweave structure of collagen, an innovative bioimaging approach combining Fast Fourier Transform (FFT) with Gabor filtering was applied [49]. Picrosirius red images were first converted to monochrome grey scale into ImageJ software, and then a 3 × 3 median filter was applied to remove photon noise generated during image acquisition. Gabor filtering was performed using ω direction values of 45° + 225°, 90° + 270°, 135° + 315°, and 0° + 180° to detect and highlight collagen fiber edges. Before FFT processing, windowing was performed on Gabor-filtered images to minimize vertical and horizontal discontinuities at the image edges that may result in artefactual lines in the frequency domain. As FFT extracts the strength of the different frequency waveforms contributing to the pixel values of Gabor-filtered cross-polar collagen images, elliptical measurements of the scatter pattern for each orientation may be used to determine a collagen orientation index (N), calculated as previously described [34].

### 2.9. Second Harmonic Generation Imaging

Collagen second harmonic generation (SHG) imaging was performed using a Zeiss multiphoton laser scanning LSM710 NLO microscope, equipped with a 20× objective (0.8 NA). A titanium:sapphire laser (Coherent Inc., Santa Clara, CA, USA) tuned to 860 nm provided the illumination light, while emitted photons were detected through a 430 ± 20 nm filter. One micron-step Z stacks of the complete section were acquired using the scan slide mode. Collagen density was quantified as the collagen-positive pixels area percentage in the thresholded image on Z-stack projections.

### 2.10. Statistical Analyses

For in vivo data, IHC, and imaging analyses, groups were compared using the non-parametric two-tailed Mann–Whitney *U* test for unpaired samples using Prism 5.0 (GraphPad Software, La Jolla, CA, USA). A Chi-squared test was used to determine a difference between the number of tumors with or without edema in each mouse group. Two-sided *p*-values < 0.05 (*) are indicated when statistical significance was reached.

## 3. Results

### 3.1. Evaluation of Endogenous Lumican Impact on Tumor Growth in ID8 Ovarian Allograft Model

**Figure 2:**
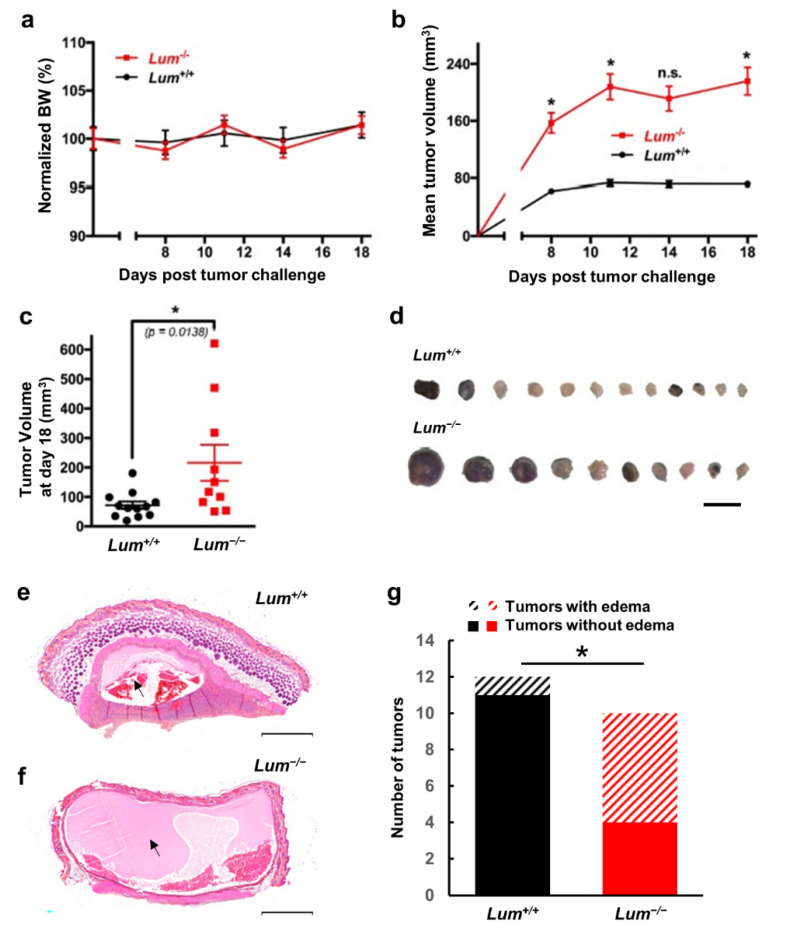
Evaluation of endogenous lumican impact on tumor growth in an ovarian allograft model. (**a**–**d**) ID8 ovarian tumor cells (2.5 × 10^5^) were s.c. inoculated in wild-type (*Lum^+/+^*) or lumican-deficient (*Lum^−/−^*) syngeneic C57BL/6J mice; (**a**) Evolution of normalized mice body weights (BW) expressed as a percentage of day 0 (mean ± SEM); (**b**) Averages of calculated tumor volumes in mm^3^ (mean ± SEM, *n* = 10–12 per group) (ns: not significant, * *p* < 0.05); (**c**) Scatter dot plot of individual calculated tumor volumes on day 18. Line, mean ± SEM (ns not significant, * *p* < 0.05); (**d**) Representative photographs of ID8 ovarian tumors s.c. allografts after tumor excision (scale bar, 1 cm). Representative images of edemas observed in HES staining of *Lum^+/+^* (**e**) and *Lum^−/−^* (**f**) tumor sections are shown (scale bar, 500 µm); (**g**) Quantification of the number of edemas observed in ovarian tumor sections of *Lum^+/+^* or *Lum^−/−^* syngeneic C57BL/6J mice (* *p* < 0.05).

After 18 days of subcutaneous inoculation of ID8 ovarian tumor cells, mice did not exhibit any adverse clinical signs, body weight (BW) loss (Figure 2a), or mortality/morbidity. In contrast, the mean tumor volume significantly increased in *Lum^−/−^* mice (Figure 2b–d). Moreover, edemas were observed in tumor sections of both groups (Figure 2e,f), but were significantly predominant in *Lum^−/−^* mice (Figure 2g, *p* = 0.02). These differences might be explained by the loss of ECM integrity due to altered collagen fibrilogenesis caused by lumican depletion, resulting in an increased tumor growth [34,40].

Alterations in stromal tissue components can inhibit or promote epithelial tumorigenesis. Lumican expression in advanced colorectal cancer with nodal metastasis was detected in 62.7% of patients, and was correlated with the spread of lymph node metastasis, the depth of tumor invasion and significantly lower survival rates of patients [50]. The expression of lumican in stromal tissues is correlated with shorter survival times of pancreatic cancer patients [51]. Another study reported that extracellular lumican enhanced the cytotoxicity of chemotherapy in pancreatic ductal adenocarcinoma cells by autophagy inhibition [52]. In lung adenocarcinoma (ADC) and squamous cell carcinoma (SCC), the expression pattern and glycosylated form of lumican in cancer cells, as well as in stromal tissue correlated with the aggressiveness of ADC and SCC [22]. Lumican is highly expressed within the stroma surrounding several solid tumors, such as lung ADC [53] and prostate cancer [54]. These pro-tumoral properties are mainly associated with lumican-mediated up-regulations of MMP-9, focal adhesion kinases (FAK), and mitogen-activated protein kinases (MAPK) [28].

In contrast, lumican has previously been shown to inhibit breast cancer migration [55,56]. Similarly, the downregulation of lumican expression in melanoma was associated with increased invasion [57]. Lumican was shown to inhibit melanoma cell migration [42,58], while promoting their adhesion [59], notably by direct interaction with α2β1 integrin [60]. Molecular processes are described as involving down-regulations of extracellular signal-regulated kinases (ERK), MMP14, and FAK [28]. These results suggest that lumican might have a similar anti-tumoral effect on ovarian cancer. Moreover, lumican was found to be transcriptionally repressed by the high-mobility group AT-hook 2 (HMGA2). The overexpression of HMGA2 confers a powerful oncogenic signal in ovarian cancers through the modulation of EMT genes [61].

### 3.2. Histological and Immunohistochemical Analysis of Ovarian Tumor Sections in Wild-Type and Lumican-Deficient Mice

**Figure 3:**
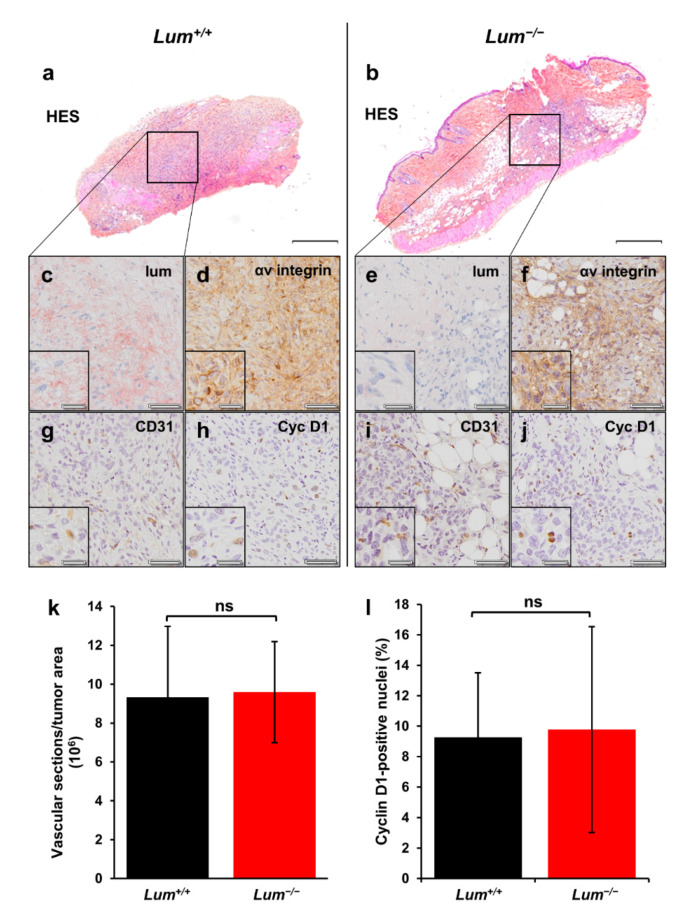
Histological and immunohistochemical analysis of ovarian tumor sections. (**a**,**b**) Example of s.c. allograft whole sections stained with HES (top panel, scale bar 500 µm) in wild-type (*Lum^+/+^*) (**a**) and lumican-deficient (*Lum^−/−^*) mice (**b**); (**c**–**j**) IHC analyses of ovarian allografts in tumors implanted in *Lum^+/+^* (**c**,**d**,**g**,**h**) and *Lum^−/−^* (**e**,**f**,**i**,**j**) mice; (**c**,**e**) Microscopic views of s.c. allograft whole sections IHC allowing visualization of lumican within tumors implanted in *Lum^+/+^* mice (**c**) while it is not detected in tumors from *Lum^−/−^* animals (**e**) (scale bar 50 µm). Insets show higher magnification (scale bar 20 µm) of stromal margin surrounding ovarian tumor allografts. Ovarian tumors from *Lum^−/−^* animals lack immunoreactive material confirming the absence of any lumican gene product; (**d**,**f**) IHC allowing visualization of αv integrin (scale bar 50 µm). Insets show higher magnification (scale bar 20 µm) of endothelial cells ovarian tumor allografts in tumors implanted in *Lum^+/+^* mice (**d**) and in tumors from *Lum^−/−^* animals (**f**); (**g**,**i**) CD31 immunostaining in tumors implanted in *Lum^+/+^* mice (**g**) and in tumors from *Lum^−/−^* animals (**i**) (scale bar 50 µm). Insets show higher magnification (scale bar 20 µm); (**h**,**j**) Cyclin D1 immunostaining in tumors implanted in *Lum^+/+^* mice (**h**) and in tumors from *Lum^−/−^* animals (**j**) (scale bar 50 µm). Insets show higher magnification (scale bar 20 µm); (**k**,**l**) Quantification of percentage of CD31-positive blood vessels (**k**) as well as relative cyclin D1-positive areas (number of positive cyclin D1 tumor cell nuclei normalized to the total number of tumor cell nuclei) (**l**) (mean ± SD, ns: not significant). The quantification of the MicroVascular Density (MVD) was based on a manual counting of full vascular sections formed by CD31-positive endothelial cells. All acquisitions were performed with a 20× magnification.

As shown in Figure 3a,b, a difference in the organization of ovarian primary tumors was observed from HES staining. In *Lum^+/+^* mice, tumors predominantly formed compact masses, while they were more scattered and diffused in *Lum^−/−^* mice. This histological observation confirms the role of lumican in maintaining the ECM architecture and, thus, controlling tumor development. The expression of endogenous lumican was verified by immunohistochemistry in *Lum^+/+^* (Figure 3c) and *Lum^−/−^* (Figure 3e) mice. As expected, lumican was detected in *Lum^+/+^* mice, while its expression was not detected in *Lum^−/−^* mice. The αv integrin subunit was previously reported to be overexpressed in ovarian cancer cells [62,63,64]. Immunohistochemical staining of this target permitted to discriminate tumor from healthy tissue in both groups (Figure 3d,f). Vascular sections present within tumor masses were quantified by immunohistochemical staining of the endothelial cell marker CD31 (Figure 3g,i). Unexpectedly, quantification did not show any difference in vessel density between *Lum^+/+^* and *Lum^−/−^* tumors (Figure 3k), while lumican was previously described to be angiostatic [58,65,66], as opposed to other SLRPs such as biglycan [28]. Lastly, cancer cell proliferation was assessed by immunohistochemical staining of the cell cycle marker cyclin D1 (Figure 3h,j). The latter staining was not statistically different between *Lum^+/+^* and *Lum^−/−^* mice groups. This was also unexpected since, as shown in Figure 2, tumors in *Lum^−/−^* mice grew significantly bigger than in *Lum^+/+^* mice. This could be explained by the ECM disorganization resulting in a higher presence of edemas in the *Lum^−/−^* group, dispersed tumor cells, and complicating the evaluation of tumor proliferative features. Indeed, the results were very heterogeneous, particularly within the *Lum^−/−^* group. Heterogeneous tumor vascularization and proliferation areas might also explain these results, as sections made from the tumor could not assess properly such features. Moreover, the presence of intra-tumoral cyclin D1-positive inflammatory cells might alter the evaluation of the immunohistochemical staining of the tumors.

### 3.3. Investigation of Intra-Tumoral Collagen Organization of Ovarian Tumor Sections in Wild-Type and Lumican-Deficient Mice

**Figure 4:**
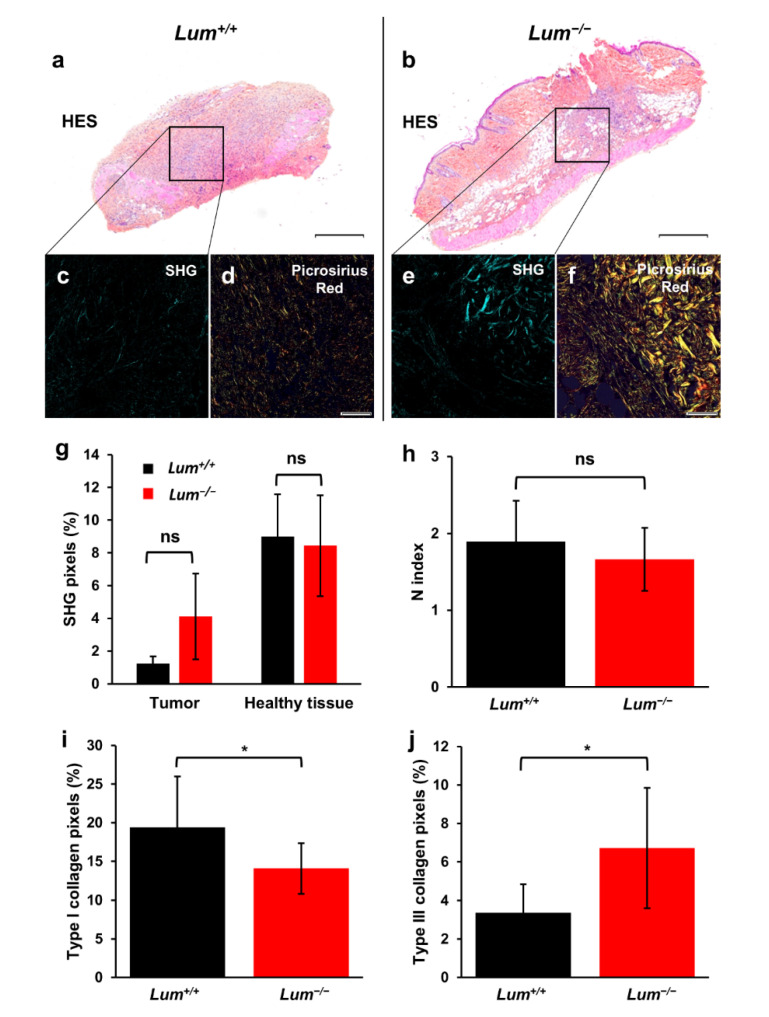
Analysis of collagen organization in ovarian tumor sections of wild-type and lumican-deficient mice. (**a**,**b**) Representative microphotographs of s.c. allograft sections stained with HES (top panel, original magnification 20×, scale bar 500 µm) in *Lum^+/+^* (**a**) and *Lum^−/−^* mice (**b**); (**c**–**f**) Picrosirius red and SHG image analyses of ovarian allografts in tumors implanted in *Lum^+/+^* mice (**c**,**d**) and in tumors from *Lum*^−/−^ mice (**e**,**f**); (**c**,**e**) Collagen SHG images from ID8 ovarian tumors (original magnification 20×); (**d**,**f**) Ovarian tumor sections stained with Picrosirius red and viewed under widefield cross-polar optics (original magnification 20×, scale bar 50 µm). Birefringence of collagen fibers allows distinction between type I (red) and type III (green) collagens; (**g**) Analysis of collagen fibers intensity by SHG in tumors and healthy tissues present in each section (mean ± SD, ns: not significant); (**h**) Analysis of tumor ECM collagen organization from images derived from Gabor filtering and FFT, processed on Picrosirius red images (mean ± SD, ns: not significant); (**i**) Quantification on Picrosirius red stained sections of the relative distribution of red pixels (corresponding to type I collagen) in tumor ECM of *Lum^+/+^* and *Lum^−/−^* sections (mean ± SD, * *p* < 0.05); (**j**) Quantification on Picrosirius red stained sections of the relative distribution of green pixels (corresponding to type III collagen) within tumors of *Lum^+/+^* and *Lum^−/−^* sections (mean ± SD, * *p* < 0.05).

In Figure 4, ECM architecture was investigated by focusing on collagen fibers organization. HES staining of *Lum^+/+^* (Figure 4a) and *Lum^−/−^* (Figure 4b) are shown once more for comparison. Firstly, SHG imaging was used to visualize collagen fibers (Figure 4c,e). Due to the presence of edemas, the number of sections in the *Lum^−/−^* group that could be analyzed by SHG was limited (*n* = 4) in order to provide a statistical evaluation. Nevertheless, *Lum^−/−^* tumors tend to exhibit more SHG marking than *Lum^+/+^* ones (Figure 4g), highlighting a higher concentration of intra-tumoral collagen that might be due to tumors infiltrating more ECM and healthy tissues in *Lum^−/−^* mice, compared to *Lum^+/+^* mice.

To pursue the characterization of intra-tumoral collagen organization, s.c. allografts sections were visualized under polarized light following Picrosirius red staining (Figure 4d,f) in order to evaluate type I and type III collagen distribution. Unexpectedly, analysis of the collagen fibers organization by processing of Picrosirius red images with Gabor filter (Figure 4h) did not show any significant difference, unlike the less organized collagen network that was previously observed in similar conditions in melanoma models [34]. This might be explained by the limited number of samples that could be analyzed with our methodology.

Pixel count calculations showed a decrease in signals arising from healthy tissue type I collagen (Figure 4i), and an increase in the intra-tumoral type III collagen (Figure 4j) in *Lum^−/−^* mice, as compared to WT. The contrast between SHG and Picrosirius red results may be due to a partly denatured type I collagen network in *Lum^−/−^* mice. Denatured collagen is detectable with Picrosirius red, but not SHG, due to the loss of a regular double helix structure, preventing emission of the SHG signal. Together, these results could be explained by an increased tumor infiltration and spreading in *Lum^−/−^* mice due to ECM disorganization.

Previous studies have shown the ability of lumican (and its derived peptides), in contrast to decorin (DCN), to inhibit MMP-14 activity in melanoma cells, where lumican directly interacted with MMP-14 [67,68,69]. Thus, lumican can maintain skin ECM integrity by inhibiting MMPs activity, and consequently melanoma progression and epithelial-mesenchymal transition (EMT). This process might likely be involved in lumican-mediated ECM alteration in the context of ovarian cancer.

### 3.4. FTIR Histopathology of Ovarian Tumor Tissues in Wild-Type and Lumican-Deficient Mice 

**Figure 5:**
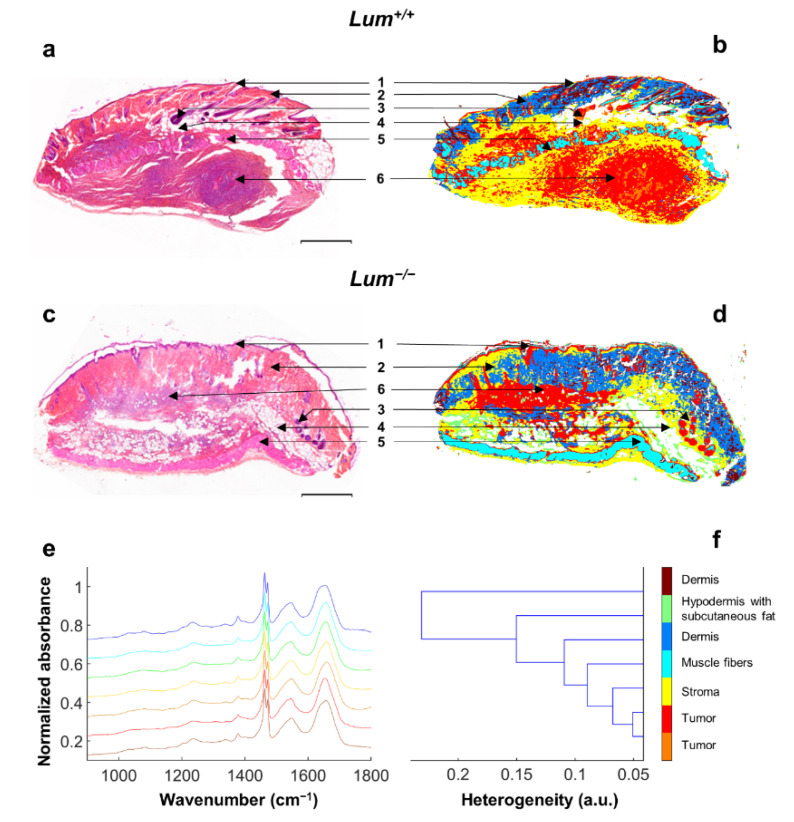
K-means clustering of FTIR spectral images of ovarian tumor sections in wild-type and lumican-deficient mice. (**a**,**c**) Example of s.c. allograft whole sections stained with HES (original magnification 20× scale bar 500 µm) in *Lum^+/+^* (**a**) and *Lum^−/−^* mice (**c**); (**b**,**d**) Representative color-coded K-means (7 classes) clustered images of tumor sections in *Lum^+/+^* (**b**) and *Lum^−/−^* mice (**d**) (1: epidermis, 2: dermis, 3: hair bulb, 4: hypodermis, 5: smooth muscle, 6: tumor); (**e**) EMSC-corrected spectra (900–1800 cm^−1^) of centroids of all seven clusters. Spectra are offset for clarity; (**f**) Dendrogram obtained as a result of hierarchical clustering showing spectral heterogeneity between the seven cluster centroids estimated by unsupervised K-means clustering of s.c. tumor infrared images. Random pseudo-colors were attributed to each cluster, while comparison to adjacent HES-stained sections allowed histological annotations of K-means subclasses.

In Figure 5, conventional histology (HES staining) and infrared spectral imaging of *Lum^+/+^* (Figure 5a,b) and *Lum^−/−^* (Figure 5c,d) mice tissue sections were compared. Using common K-means clustering with seven classes, all main histological structures could be identified, namely the epidermis, dermis, hypodermis, muscle fibers, and ovarian tumor. However, it can be noted that epidermis, hair bulbs, and tumor masses are represented by the same cluster. An interesting observation between *Lum^+/+^* and *Lum^−/−^* tissue sections is that the same morphological features were detected, with, however, a difference in tumor cells distribution, which is more compact in *Lum^+/+^*. This is concordant with what was observed in HES-stained images in Figure 3. Nevertheless, no significant differences in cluster distribution could be identified between *Lum^+/+^* and *Lum^−/−^* mice. The different clusters are represented by their centroid spectra (Figure 5e), and the grouping of spectra are shown in the dendrogram (Figure 5f). Each class represents an anatomical structure, except for tumor masses and the dermis, which are each represented by two classes. For example, tumor masses are represented by the red and orange clusters, while the dermis is represented by the brown and blue clusters.

**Figure 6:**
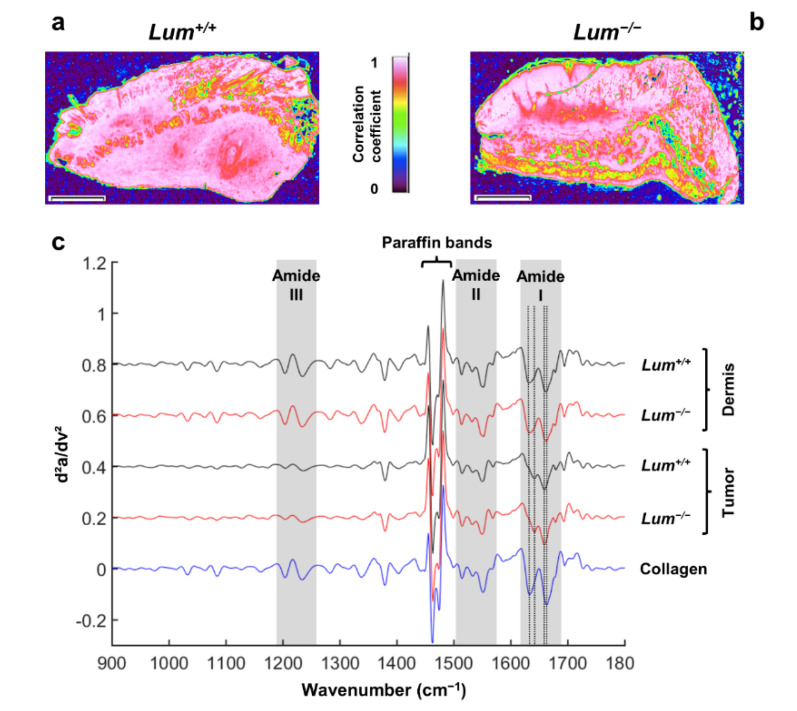
Correlation maps using type I collagen reference spectrum. (**a**,**b**) Type I collagen spectral correlation images of *Lum^+/+^* (**a**) and *Lum^−/−^* (**b**) tissue sections (scale bar 500 µm). Original images were each correlated with a pure type I collagen spectrum. Provided scale indicates the degree of correlation from 0 (black, not correlated) to 1 (white, completely correlated). (**c**) Comparison between type I collagen second derivative spectrum (blue line) with second derivative spectra taken randomly from the dermis and tumors of *Lum^+/+^* (black lines) and *Lum^−/−^* (red lines) mice skin tissues. Second derivative spectra are offset for clarity. The corresponding collagen-characteristic bands of Amide I, II, and III are highlighted. Dotted lines in the Amide I band indicate 1633, 1641, 1658, and 1662 cm^−1^ wavenumbers, respectively.

Type I collagen being a major component of the ECM, its distribution was assessed in *Lum^+/+^* and *Lum^−/−^* mice tissues by using a reference type I collagen spectrum to construct correlation images. These are displayed in Figure 6a,b along with a correlation scale. The results showed that collagen appeared denser in the dermis and in the tumor periphery in *Lum^+/+^* mice. In contrast, in *Lum^−/−^*, collagen distribution was looser, especially at the tumor site, due to ECM disorganization, as also observed in SHG images. The lack of a clear difference in collagen correlation between *Lum^+/+^* and *Lum^−/−^* mice might be explained by the capacity of FTIR to detect both well organized and denatured collagen [70], as discussed above. To further analyze collagen modifications, second derivative spectra of *Lum^+/+^* and *Lum^−/−^* tumors and dermis were compared with a reference type I collagen spectrum (Figure 6c). The second derivative profile of native collagen was comparable to both dermis samples from *Lum^+/+^* and *Lum^−/−^* mice. In contrast, second derivative profile of native collagen exhibited differences with both tumors from *Lum^+/+^* and *Lum^−/−^* mice, specifically in the Amide I and III regions. The main impacted Amide I bands are identified at 1641 cm^−1^ (1633 cm^−1^ in native collagen) and 1658 cm^−1^ (1662 cm^−1^ in native collagen) in the tumors. The shifts are respectively of +8 cm^−1^ and −4 cm^−1^. These bands relate to protein secondary structures, indicating a change in structural conformation of type I collagen, the major component of the ECM. In addition, the absorption bands in the 1000–1100 cm^−1^ region are more prominent in collagen and dermis spectra, compared to the tumor spectra. However, this analysis did not reveal any significant difference between *Lum^+/+^* and *Lum^−/−^* mice, both in the dermis and tumor regions.

## 4. Conclusions

Overall, the present report is the first to highlight the major role of lumican in the maintenance of the extracellular matrix integrity in the context of ovarian cancer, showing its inhibitory role in primary ovarian tumor allografts growth. Thanks to a multimodal approach, combining histopathology, immunohistochemistry, and three optical imaging techniques, the alteration of collagen organization could be demonstrated in tumors from lumican-deficient mice. This disorganization was associated with a significant increase in tumor growth and edema formation within the tumors. Non-invasive methods such as FTIR imaging represent potential diagnostic techniques for detection of ovarian tumors at early stages. Moreover, these techniques are promising in evaluating ECM integrity, leading to a more appropriate treatment to target cancer cells while preserving ECM structure.

## Data Availability

The data presented in this study are available on request from the corresponding author.
